# Multi-features taxi destination prediction with frequency domain processing

**DOI:** 10.1371/journal.pone.0194629

**Published:** 2018-03-22

**Authors:** Lei Zhang, Guoxing Zhang, Zhizheng Liang, Ekene Frank Ozioko

**Affiliations:** 1 School of Computer Science and Technology, China University of Mining and Technology, Xuzhou, Jiangsu, China; 2 School of Mathematical, Physics and Computational Science, University of Reading, Reading, United Kingdom; Beihang University, CHINA

## Abstract

The traditional taxi prediction methods model the taxi trajectory as a sequence of spatial points. It cannot represent two-dimensional spatial relationships between trajectory points. Therefore, many methods transform the taxi GPS trajectory into a two-dimensional image, and express the spatial correlations by trajectory image. However, the trajectory image may have noise and sparsity according to trajectory data characteristics. So, we import image frequency domain processing to taxi destination prediction to reduce noise and sparsity, then propose multi-features taxi destination prediction with frequency domain processing (MTDP-FD) method. Firstly, we transform the spatial domain trajectory image into frequency-domain representation by fast Fourier transform and reduce the noise of the trajectory images. Convolutional Neural Network (CNN) is adapted to extract the deep features from the processed trajectory image as CNN has a significant learning ability to images. Recurrent Neural Network (RNN) is adapted to predict the taxi destination as multiple hidden layers of RNN can store dependencies between input data to achieve better prediction. The deep features of the trajectory images are combined with trajectory metadata, trajectory data to act as the input to RNN. The experiments based on the taxi trajectory dataset of Porto show that the average distance error of MTDP-FD is reduced by 0.14km compared with the existing methods, and the GTOHL is the best combination of data and features to improve the prediction accuracy.

## Introduction

With the popularization and development of vehicle GPS technology, a large number of GPS trajectory data are generated in taxi driving. The accumulation GPS trajectory data makes it possible to predict taxi destination. Taxi destination refers to the destination of a passenger. If the taxi is vacant, the trajectories will not be recorded and we will not take the taxi driver’s destination as taxi destination. The main purpose of predicting destination is resource allocation, traffic analysis, and improving transportation networks [1]. The accurate and efficient prediction of taxi destination can not only provide personalized navigational services for the taxi, but also play an important role in urban intelligent planning, reasonable regulation and allocation of traffic resources [2, 3]. On the one hand, destination prediction can match passengers with the nearest available taxi to minimize the wait-time to increase customer satisfaction. Particularly, there is often a taxi whose current ride will end near or exactly at a requested pick up location from a new rider. It is beneficial for taxi companies to organize the taxi fleet to improve services and profits. On the other hand, destination prediction can help to forecast traffic conditions and automatically plan the best path to avoid traffic jam and provide intelligent navigation service.

Mining the effective information of taxi GPS data is widely used in all aspects, such as, taxi customer searching behaviors for improving the system services [4], understanding Origin—Destination distribution of taxi trips for improving effects of transportation planning and enhancing quality of taxi services [5], map-matching for determining vehicle position on the road [6], uncovering urban human mobility for city and transportation planning [7], revealing intra-urban travel patterns and service ranges for addressing many urban sustainability challenges [8] and so on. Among them, taxi destination prediction is a very hot topic. The existing taxi destination prediction methods can be divided into two families: statistical methods and neural networks [9].

Statistical methods are widely used in taxi trajectory destination prediction. Xue AY et al. [10, 11] identified the data sparsity problem in destination prediction and proposed a novel Sub-Trajectory Synthesis (SubSyn) algorithm to address this problem. They used a low-order Markov model to infer potential destinations. We have proposed a sparse trajectory prediction method based on entropy estimation (STP-EE) [12] and an entropy-based sparse trajectories prediction method enhanced by matrix factorization (ESTP-MF) [13] to resolve the data sparsity problem. STP-EE uses trajectory entropy estimation to evaluate trajectory’s regularity and chooses trajectories with lower entropy values to reduce the number of abnormal trajectories. And ESTP-MF adopts matrix factorization to infer transition probabilities of the missing regions from corresponding existing elements in the transition probability matrix. Besse PC et al. [14] proposed a data-driven method to predict the final destination of vehicle trips using a statistic learning procedure. They modelled the main paths by clustering trajectories and modelled main traffic flow patterns within each trajectory’s cluster by a mixture of 2d-Gaussian distributions. Although these statistical methods have been widely used in the prediction of taxi destination, these methods simply import the GPS trajectory points into the statistical models and ignore the important spatiotemporal features of the trajectory data. Zou Y et al [15] took into account spatial and temporal information of data for travel time prediction, the method can be used to obtain reliable short-term prediction of travel time, and the results are more robust than the traditional methods. So the spatiotemporal features contain some important information and play a vital role. Therefore, it is not the best choice to use the statistical methods which ignored the spatiotemporal features in the taxi destination prediction.

Artificial neural networks (ANN) are used in taxi destination prediction because of its ability of dealing with multi-dimensional data, implementation flexibility, generalizability, and strong forecasting power [9]. There are a variety of artificial neural networks in the prediction. Ma X et al. proposed a novel architecture of Long Short-Term Neural Network (LSTM) [16] and a Deep Convolutional Neural Network (CNN) [17] for traffic speed prediction. The LSTM model is to capture nonlinear traffic dynamic for traffic speed prediction, and this method can achieve the best prediction performance in terms of both accuracy and stability. The CNN model is to learn traffic as images and predict large-scale, network-wide traffic speed with a high accuracy. Tang J et al [18] improved the Fuzzy Neural Network in traffic speed prediction, this method can consider the periodic pattern and demonstrate advantages over other models with smaller predicted errors and slow raising rate of errors. De Brébisson A et al. [19] adopted a variety of neural network models including Recurrent Neural Network (RNN) and Long Short-Term Memory Network (LSTM) to predict the taxi destination and gained good results. Moore J et al. [20] used RNN to predict the destination of user trajectory and compared with the results that predicted by LSTM. Endo Y et al. [21] applied RNN to predict the destination from the user’s partial moving trajectory. These methods import trajectory data into the neural network model according to the time sequence. The temporal features between trajectory data are preserved in this way. However, these methods ignored the spatial relationships between the trajectory data among the GPS trajectory points.

In order to explore spatiotemporal information in trajectory data, Endo Y et al. [22, 23] firstly proposed a method that transformed trajectory data into trajectory images while maintaining effective spatiotemporal information. This method achieved good results in trajectories classification. Lv J et al. [24] allocated the image pixels according to three different classes of the initial points, middle points and end points. Then, they extracted the deep features from the image by using Convolutional Neural Networks (CNN). At last, they used the deep features to predict the taxi destination. The advantages of the above methods are shown as follows: (1)Trajectory data are transformed into trajectory images to ensure the temporal and spatial relationships between trajectory data. (2)High-level features can be automatically extracted from trajectory images by deep learning. However, the transformed trajectory images are inaccurate due to the sparsity of trajectory data. There is very large noise in the trajectory image. Inaccurate trajectory images lead to the inaccuracy of extracted deep features.

In the field of image processing, many methods combine the spatial domain with frequency domain to process images as the frequency domain features can well represent the varying degree of images and remove the noises of the images [25, 26]. In order to reduce the impact of noises in trajectory images and improve the accuracy of taxi destination prediction, we propose multi-features taxi destination prediction with frequency domain processing method which is called MTDP-FD. MTDP-FD transforms the spatial domain trajectory images into the frequency domain representation of images. Frequency domain processing is firstly introduced into the taxi destination prediction. Furthermore, the CNN is applied to extract the deep features from trajectory images and frequency domain representation of images, which not only maintains the temporal and spatial relationships of the trajectory data, but also reduces the noise of the trajectory image. So, it improves the prediction accuracy. MTDP-FD combines the deep features that extracted by CNN with data to act as the input of the RNN to predict the taxi destination. Finally, we explore which ways of features and data combination can significantly improve the prediction accuracy through experiments.

## MTDP-FD framework

**Fig 1:**
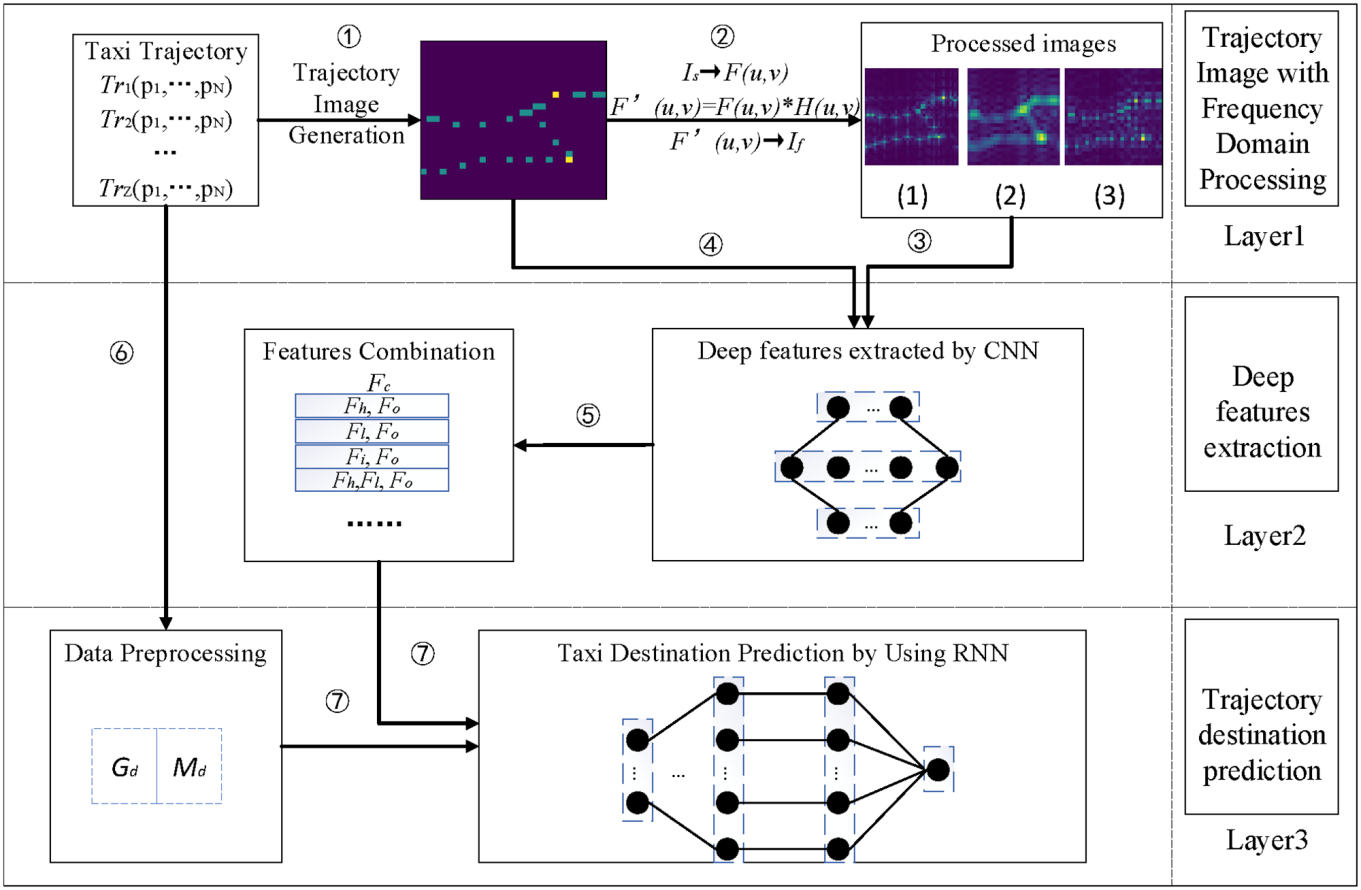
MTDP-FD framework.

Firstly, MTDP-FD converts a raw trajectory data structure into an image data structure. Secondly, the trajectory image is processed in frequency domain representation. CNN are used to extract the deep features from processed images. Then, deep features derived from the trajectory image are combined with trajectory metadata, trajectory data to act as the input of the RNN to predict the taxi destination. MTDP-FD framework is shown in Fig 1.

The steps of MTDP-FD are described as follows:

(1)Step1: To strengthen temporal and spatial relationships between trajectory data, we transform each trajectory into a two-dimensional trajectory image *I*_*s*_.

(2)Step2: We convert the trajectory image to frequency domain matrix using fast Fourier transformation and reduce the noise of the trajectory image. Then, the processed frequency domain matrix is transformed to three spatial domain images using inverse transformation based on different filter functions.

(3)Step3: We obtain the three deep features *F*_*h*_, *F*_*l*_, *F*_*i*_ of the three processed images by CNN as CNN has a significant learning ability to images and can extract important features from images.

(4)Step4: We extract the deep features from the original spatial domain trajectory images by CNN, and obtain the original image deep features which called *F*_*o*_.

(5)Step5: We combine *F*_*h*_, *F*_*l*_, *F*_*i*_ with *F*_*o*_ as a one-dimensional feature sequence, and obtain the combination feature which is called *F*_*c*_.

(6)Step6: We take the first *k* points and last *k* points of the trajectory data to form a fixed length trajectory sequence *G*_*d*_, and the corresponding metadata *M*_*d*_ is also taken out.

(7)Step7: We combine the *G*_*d*_, *M*_*d*_ with *F*_*c*_ to act as the input of RNN to predict the taxi destination.

## Frequency domain processing of trajectory image

The trajectory data is transformed into two-dimensional trajectory images, which ensures the temporal and spatial relationships between the trajectory data. The trajectory images are converted from spatial domain to frequency domain to be able to reduce the noises of the image and alleviate the sparsity.

### Transforming trajectory data into trajectory image

Accurate and reasonable transformation of trajectory data into trajectory images can keep the temporal and spatial relationships between trajectory data. Morever, proper handling of the relationships between the number and value of pixels in the trajectory images can alleviate the data sparsity problem.

**Fig 2:**
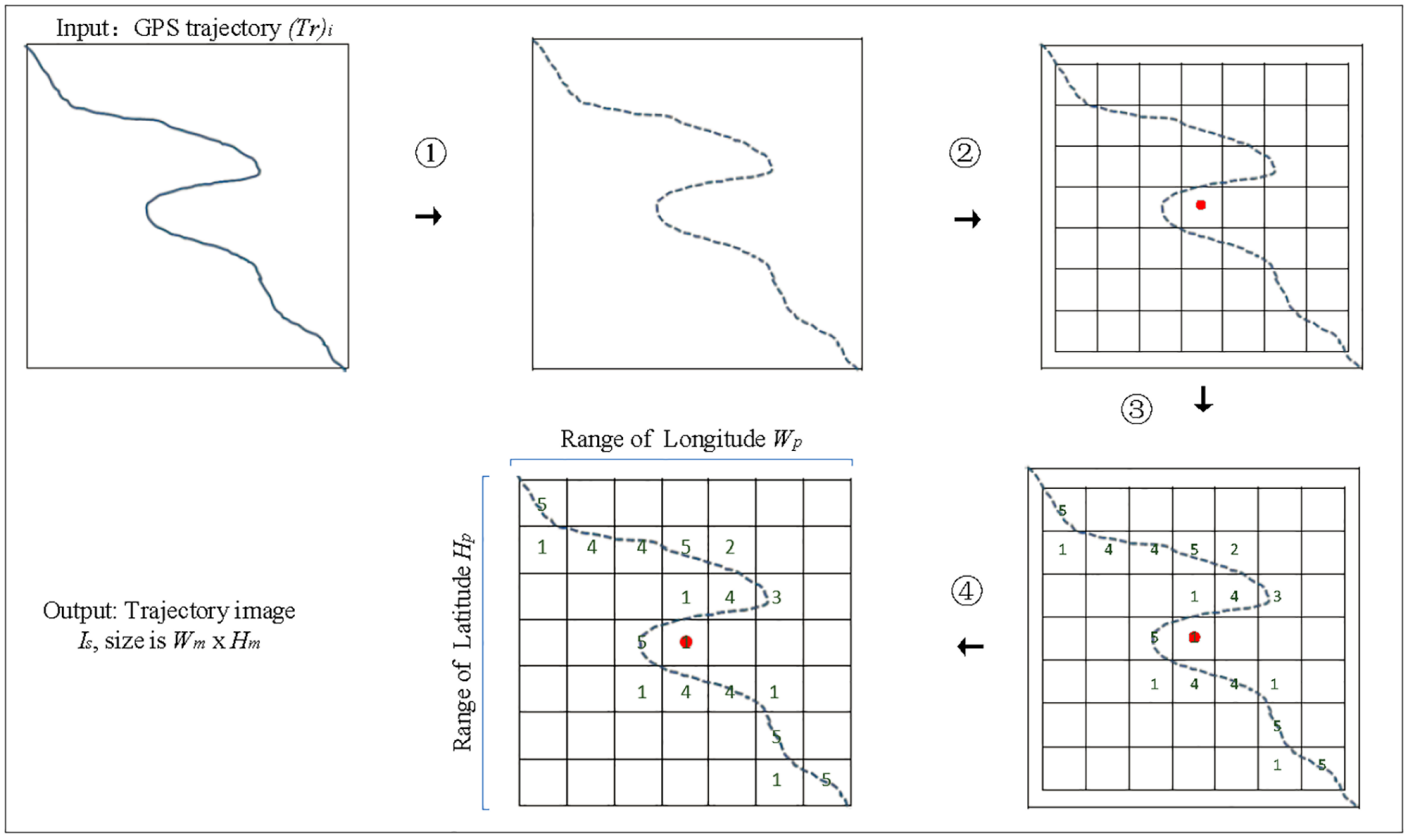
Transform trajectory data into trajectory images. The input is a GPS trajectory sequence *T*_*r*_, and the output is a two-dimensional trajectory image *I*_*s*_.

Taxi trajectories are composed of the trajectory data and the basic features of trajectory data, such as taxi number, travel date type and so on. Trajectory data is a sequence of continue GPS points. We use (*T*_*r*_ = (*p*_1_, *p*_2_, …, *p*_*i*_, …, *p*_*N*_)) to represent a taxi trajectory, where *N* denotes the number of GPS points. Each GPS point is expressed by a tuple of *p*_*i*_ = (*lat*_*i*_, *lng*_*i*_, *t*_*i*_), where *lat*_*i*_ denotes latitude, *lng*_*i*_ denotes longitude and *t*_*i*_ denotes time index. MTDP-FD transforms each trajectory data into trajectory image, and the detailed process is shown in Fig 2.

(1)Step1: Trajectory sampling. A fixed interval *T* is used to evenly sample GPS points from *T*_*r*_. The purpose of trajectory sampling is to avoid that short interval will lead to a long stay. The sampling process can be written as:

$$\begin{array}{c} T_{r}^{{}^{\prime }}=sample\left(T_{r},T\right) \end{array}$$ (1)

where $T_{r}^{{}^{\prime }}$ means a sampled trajectory. If the next GPS point is not obtained after just *T*, we sample the nearest time GPS point.

(2)Step2: Computing the center of the trajectory image and image size. We let the center of the GPS trajectory as the center of the trajectory image. GPS trajectory center can be written as:

$$\begin{array}{c} center_{lng}=\frac{1}{|T_{r}^{{}^{\prime }}|}\sum_{j=1}^{|T_{r}^{{}^{\prime }}|}p_{j}.lng \end{array}$$ (2)

$$\begin{array}{c} center_{lat}=\frac{1}{|T_{r}^{{}^{\prime }}|}\sum_{j=1}^{|T_{r}^{{}^{\prime }}|}p_{j}.lat \end{array}$$ (3)

where $|T_{r}^{{}^{\prime }}|$ means the number of GPS points after trajectory sampling, *p*_*j*_.*lng* and *p*_*j*_.*lat* represent the longitude and latitude of each GPS point in $T_{r}^{{}^{\prime }}$.

While determining the image size, firstly, we define a rectangular target area of *W*_*p*_ × *H*_*p*_. The *W*_*p*_ and *H*_*p*_ denote ranges of the longitude and latitude, respectively. Then, the rectangular area is divided into *W*_*m*_ × *H*_*m*_ grids and each grid corresponds to each pixel of the trajectory image. The *W*_*m*_ and *H*_*m*_ denote width and height of the image.

(3)Step3: Determining the value of each pixel. The value of each pixel is determined according to the number of trajectory points in each grid. *I*(*x*, *y*) represents the value of each pixel and 0 ≤ *x* ≤ *W*_*m*_ − 1, 0 ≤ *y* ≤ *H*_*m*_ − 1.

(4)Step4: A two-dimensional trajectory image *I*_*s*_ is obtained, and the size of *I*_*s*_ is *W*_*m*_ × *H*_*m*_.

### Frequency domain processing process of trajectory image

Frequency domain of trajectory image represents the varying degree of the image and frequency domain processing can remove the noise of the image and alleviate the data sparsity problem.

**Fig 3:**
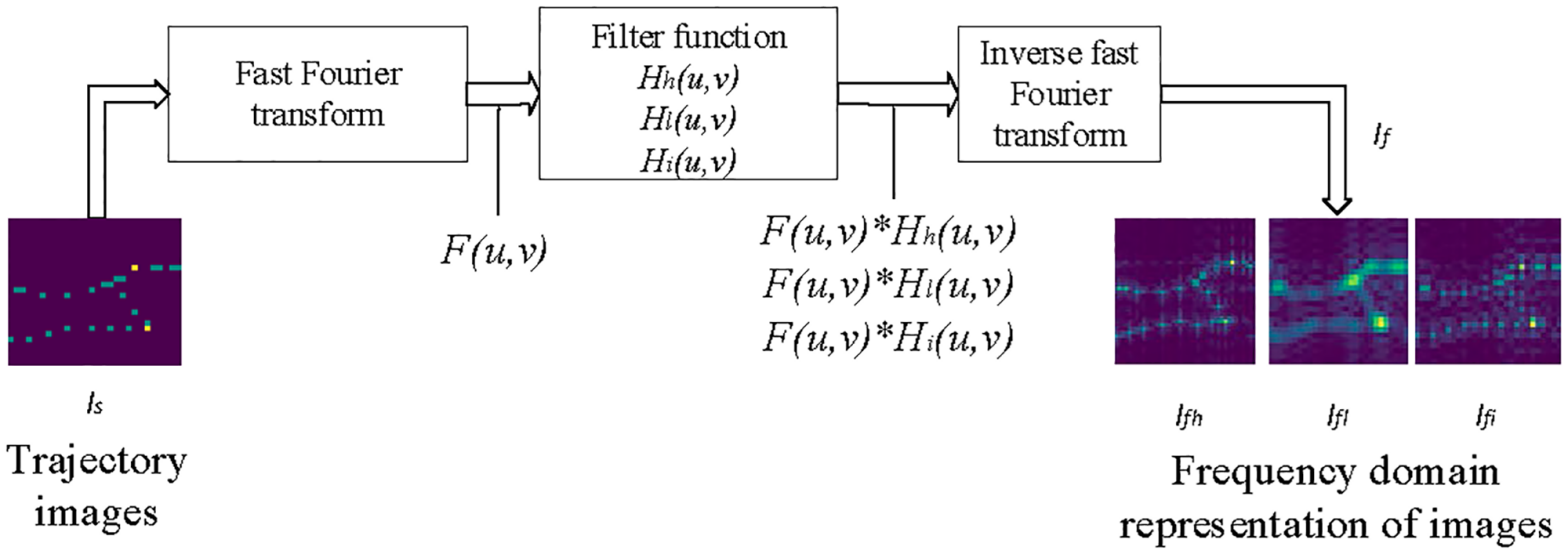
Frequency domain processing of trajectory image.

We use fast Fourier transform [26] to transform the trajectory image into the frequency domain representation. Frequency domain processing of trajectory image is shown in Fig 3.

Firstly, the trajectory image *I*_*s*_ is transformed into a frequency domain matrix *F*(*u*, *v*) by fast Fourier transform as shown in Eq 4.

$$\begin{array}{c} F\left(u,v\right)=\sum_{x=0}^{W_{m}-1}\sum_{y=0}^{H_{m}-1}I_{s}\left(x,y\right)e^{-j2\pi (ux/W_{m}+vy/H_{m})} \end{array}$$ (4)

where *u* = 0, 1, 2, …, *W*_*m*_ − 1, *v* = 0, 1, 2, …, *H*_*m*_ − 1. *W*_*m*_ and *H*_*m*_ are the width and height of *I*_*s*_. The absolute value of *F*(*u*, *v*) is taken as the amplitude feature of the frequency domain image.

Secondly, filtering in the *F*(*u*, *v*) can highlight the relevant information of the image and reduce the noise. The filtering process can be written as:

$$\begin{array}{c} F^{{}^{\prime }}\left(u,v\right)=F\left(u,v\right)*H\left(u,v\right) \end{array}$$ (5)

where *H*(*u*, *v*) is the filter function, *F*′ (*u*, *v*) is the filtered matrix. We choose 3 different filter functions including high-pass filter, low-pass filter and band-pass filter in frequency domain processing.

Lastly, we transform the filtered matrix *F*′ (*u*, *v*) to spatial domain image *I*_*f*_ which called frequency domain representation of image. The inverse transformation can be written as:

$$\begin{array}{c} I_{f}=\frac{1}{W_{m}H_{m}}\sum_{u=0}^{W_{m}-1}\sum_{v=0}^{H_{m}-1}F^{{}^{\prime }}\left(u,v\right)e^{j2\pi (ux/W_{m}+vy/H_{m})} \end{array}$$ (6)

where *x* = 0, 1, 2, …, *W*_*m*_ − 1, *y* = 0, 1, 2, …, *H*_*m*_ − 1.

### Frequency domain processing methods

We consider 3 kinds of filter functions: high-pass filter, low-pass filter and band-pass filter.

(1)High frequency representation of images *I*_*fh*_

High-pass filter is used to remove low-frequency information. The edge gray level of trajectory changes sharply, and only high frequency information is left after the high-pass filter. So the high-pass filter can extract the contour information and the shape of the trajectory. By this filtering, the noise in the trajectory image is reduced. High-pass filter is written as:

$$\begin{array}{c} H_{h}\left(u,v\right)=\{\begin{array}{ccc} 1 & & D\left(u,v\right)\geq D_{0}\\ 0 & & D\left(u,v\right)<D_{0} \end{array} \end{array}$$ (7)

where *D*(*u*, *v*) represents the distance from the point (*u*, *v*) to filter center. *D*_0_ is a nonnegative parameter corresponding to a high-pass filter.

(2)Low frequency representation of images *I*_*fl*_

Low-pass filter is used to remove high-frequency information from the image. It can blur the contour and strengthen the internal relations of the trajectory image to alleviate the trajectory sparsity. Low-pass filter is written as:

$$\begin{array}{c} H_{l}\left(u,v\right)=\{\begin{array}{ccc} 1 & & D\left(u,v\right)\leq D_{1}\\ 0 & & D\left(u,v\right)>D_{1} \end{array} \end{array}$$ (8)

where *D*(*u*, *v*) also represents the distance from the point (*u*, *v*) to filter center. *D*_1_ is a nonnegative parameter corresponding to a low-pass filter.

(3)Intermediate frequency representation of images *I*_*fi*_

Band-pass filter can preserve partial high frequency and partial low frequency in the image, and the number of reservations is determined according to the parameters in the filter. Band-pass filter is written as:

$$\begin{array}{c} H_{i}\left(u,v\right)=\{\begin{array}{ccc} 0 & & D\left(u,v\right)<D_{2}\\ 1 & & D_{2}\leq D\left(u,v\right)\leq D_{3}\\ 0 & & D\left(u,v\right)>D_{3} \end{array} \end{array}$$ (9)

Where *D*_2_ and *D*_3_ are the parameters corresponding to a band-pass filter, and *D*2 < *D*3.

After frequency domain processing, we obtain three kinds of frequency domain representation of images, including *I*_*fh*_, *I*_*fl*_ and *I*_*fi*_. And the size of *I*_*fh*_, *I*_*fl*_ and *I*_*fi*_ is also *W*_*m*_ × *H*_*m*_ because frequency domain processing does not change the size of image.

## Deep features extraction from trajectory image by CNN

CNN has the obvious advantages in deep features extraction of trajectory images. Firstly, CNN features extraction is locally connected between layers. A neuron in the posterior layer is connected to some neurons in the ahead layer. It strengthens the connection between local regions to make CNN more efficient in features extraction. Secondly, the parameters of CNN in the training process can be reduced to the maximum extent by pooling operation. At the same time, the aggregated statistics of the features at different locations ensure that the most important features can be preserved.

### The process of extracting deep features from trajectory image

**Fig 4:**
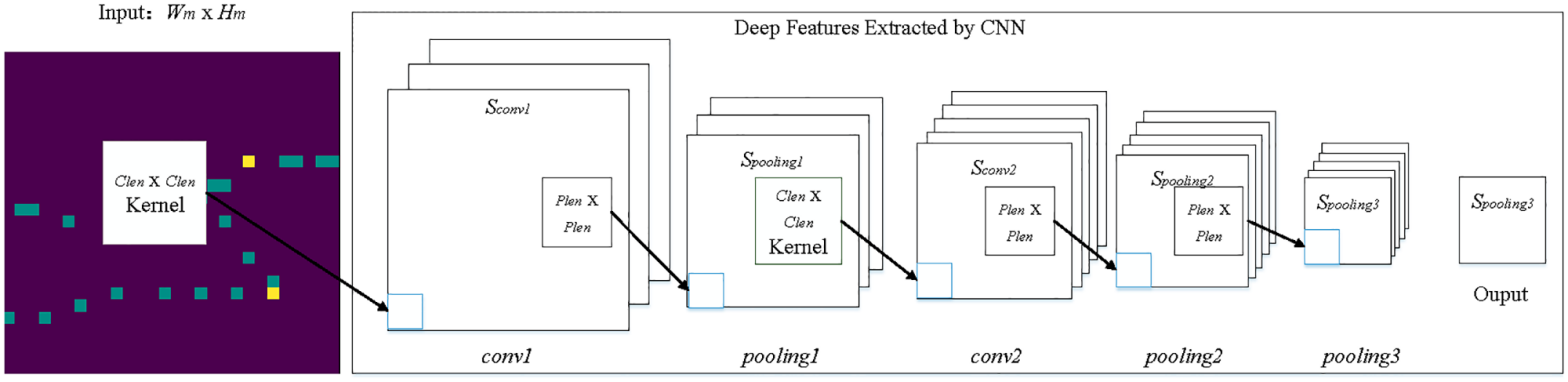
Process of extracting deep features from trajectory image.

The process of extracting deep features by CNN from trajectory image is shown in Fig 4.

Step1: We uses *I* to represent the input trajectory images which include *I*_*s*_, *I*_*fh*_, *I*_*fl*_ and *I*_*fi*_. And the size of each trajectory image is *W*_*m*_ × *H*_*m*_.

Step2: We have the first-time convolution of the trajectory images, and the size of convolution kernel is *C*_*len*_ × *C*_*len*_. The first-time convolution process can be written as:

$$\begin{array}{c} conv1=\sigma (I\circ w_{1}+b_{1}) \end{array}$$ (10)

where *σ* is the activation function RELU [27], *w*_1_ is a convolution kernel and ∘ is convolution operation. To capture as many features as possible, we set convolution stride *C*_*stride*_ is 1, *b*_1_ is the bias. The ∘ and *C*_*stride*_ are introduced in section 4.2. After the first-time convolution, the length and width of the *conv*1 *S*_*conv*1_ are ⌈(*W*_*m*_ − *C*_*len*_ + 1)/*C*_*stride*_⌉.

Step3: We have the first-time pooling to simplify the feature extraction model. The size of pooling is *P*_*len*_ × *P*_*len*_, and the first-time pooling process can be written as:

$$\begin{array}{c} pooling1=pool(conv1) \end{array}$$ (11)

where *pool* means pooling operation which introduced in section 4.2. After the first-time pooling, the length and width of the *pooling*1 *S*_*pooling*1_ are ⌈*S*_*conv*1_/*p*_*len*_⌉.

Step4: We have the second-time convolution of *pooling*1, and the size of convolution kernel is also *C*_*len*_ × *C*_*len*_. The second-time convolution process can be written as:

$$\begin{array}{c} conv2=\sigma (pooling1\circ w_{2}+b_{2}) \end{array}$$ (12)

where *σ* is also the activation function RELU, *w*_2_ is a convolution kernel and ∘ is also the convolution operation. To capture as many features as possible, we also set convolution stride *C*_*stride*_ is 1, *b*_2_ is the bias. After the second-time convolution, the length and width of the *conv*2 *S*_*conv*2_ are ⌈(*S*_*pooling*1_ − *C*_*len*_ + 1)/*C*_*stride*_⌉.

Step5: We have the second-time pooling to simplify the feature extraction model. The size of pooling is also *P*_*len*_ × *P*_*len*_, and the second-time pooling process can be written as:

$$\begin{array}{c} pooling2=pool(conv2) \end{array}$$ (13)

where pool means pooling operation which introduced in section 4.2. After the second-time pooling, the length and width of the *pooling*2 *S*_*pooling*2_ are ⌈*S*_*conv*2_/*p*_*len*_⌉.

Step6: In order to simplify the calculation and extract deeper trajectory features, we have to do the third-time pooling. The size of pooling is also *P*_*len*_ × *P*_*len*_, and the third-time pooling process can be written as:

$$\begin{array}{c} pooling3=pool(pooling2) \end{array}$$ (14)

After the third-time pooling, the length and width of the *pooling*3 *S*_*pooling*3_ are ⌈*S*_*pooling*2_/*p*_*len*_⌉.

We take *pooling*3 as the deep features. And original trajectory image features *F*_*o*_, low-frequency features *F*_*l*_, high-frequency features *F*_*h*_ and Intermediate-frequency features *F*_*i*_ can be extracted from the *I*_*s*_, *I*_*fl*_, *I*_*fh*_ and *I*_*fi*_. These features include not only the spatiotemporal features of trajectories, but also some important hidden features. MTDP-FD takes the extracted deep features as supplement or enhancement of the trajectory, and transforms the deep features into a one-dimensional sequence while using these features. In the prediction, trajectory data, trajectory metadata and this sequence are combined as input to RNN.

### Activation function, convolution and pooling

Each layer of CNN needs activation functions, the roles of activation function are as follows: (1)Adding nonlinear elements to CNN, which makes it easier for CNN to deal with complex situations in the process of extracting features. (2)Controlling the output of each convolution layer to a certain range, which is convenient for training.

In the feature extraction process, MTDP-FD uses the RELU as activation function. RELU can be written as:

$$\begin{array}{c} f\left(x\right)=\{\begin{array}{ccc} x & & x\geq 0\\ 0 & & otherwise \end{array} \end{array}$$ (15)

**Fig 5:**
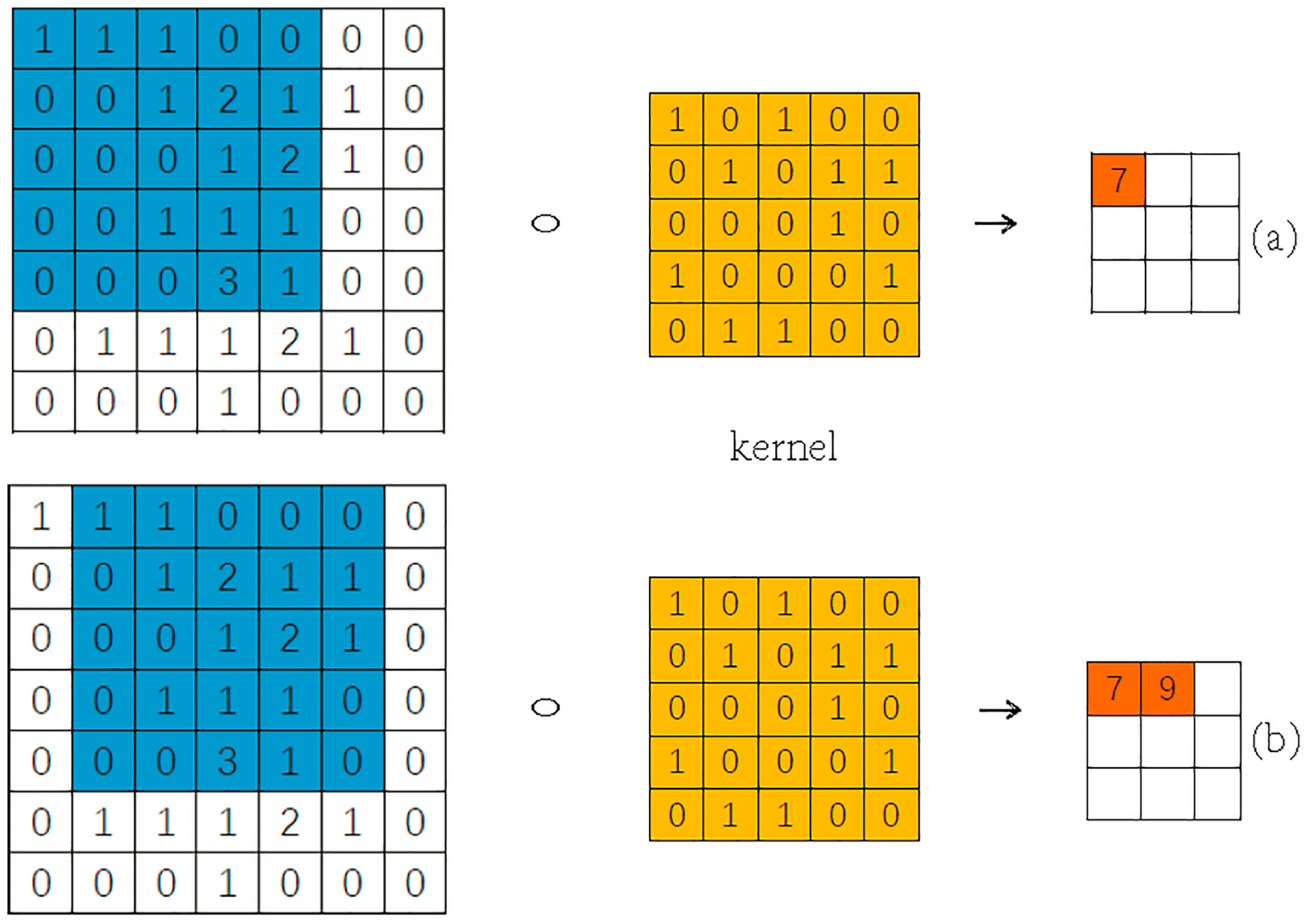
The processes of convolution. (a) and (b) are two processes of convolution.

**Fig 6:**
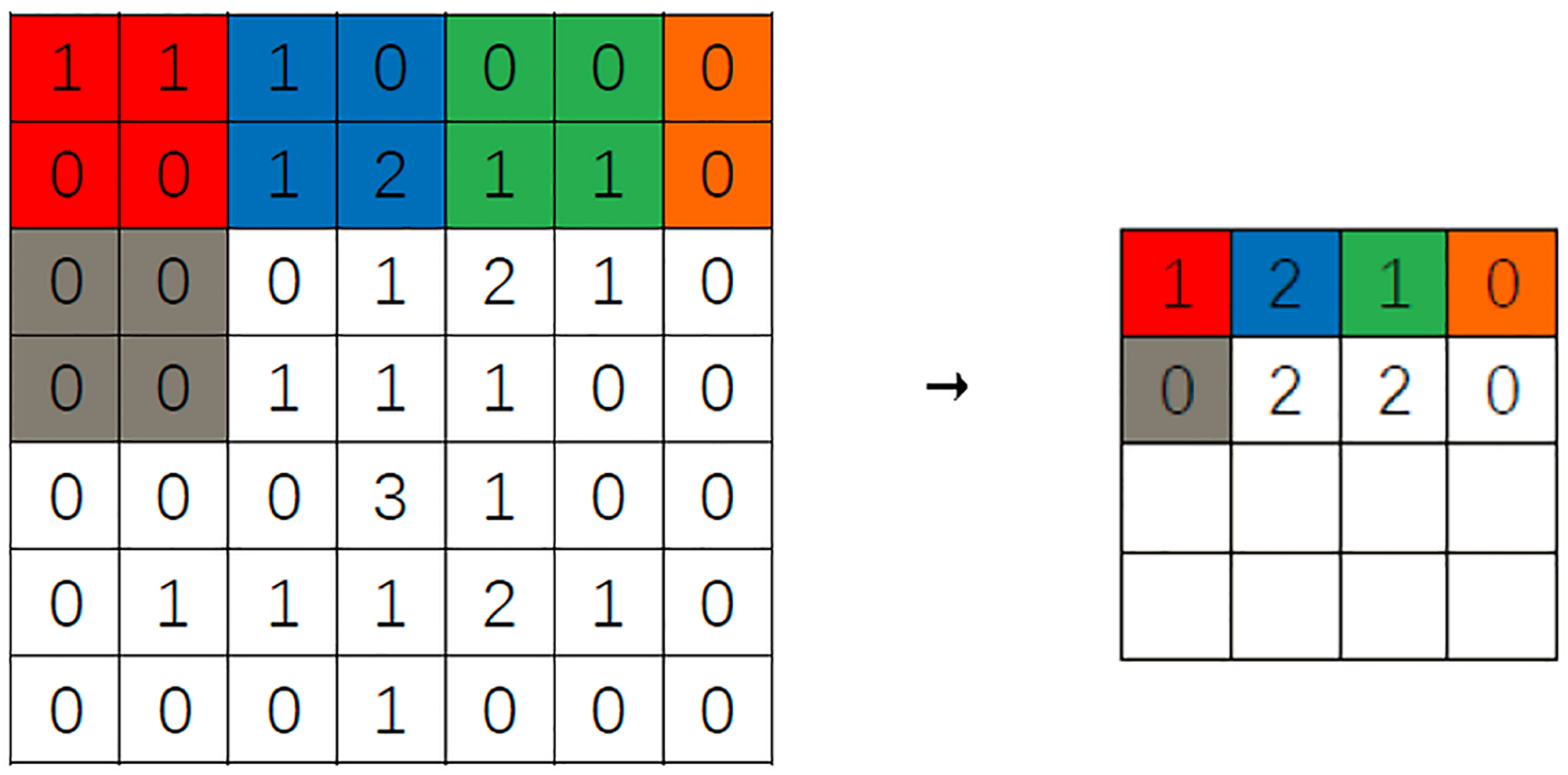
Max pooling.

Convolution and pooling are two most important processes of CNN, both of which are carried out in two-dimensional space. Suppose we need to convolute and pool the 7 × 7 size image, these processes are shown in Figs 5 and 6.

In Fig 5, we set the size of the convolution kernel is 5 × 5, and the convolution stride of 1 indicates that each convolution can only move 1 grids on length or width. The Fig 5 shows the two processes of convolution. After the convolution is completed, the length and width of image are ⌈(7 − 5 + 1)/1 = 3⌉.

There are several kinds of pooling methods, where we use the Max Pooling (take the maximum value in the pooling size) to highlight the most important features.

In Fig 6, we set the pooling size is 2 × 2, and we select the maximum value of 2 × 2 points, this method is called Max Pooling. When the pooling is completed, the length and width of image are ⌈7/2⌉ = 4. Convolution and pooling operation aggregate the features of different positions of the trajectory images, and reduces the size of the training data, and extracts the deep features of the trajectory image.

## Taxi destination prediction by RNN

We use Recurrent Neural Network (RNN) to predict taxi destination in MTDP-FD. The advantages of RNN in the taxi destination prediction are described as follows:

(1)The hidden layers of RNN can store the dependencies between trajectory data, thus ensuring the accuracy of prediction.

(2)RNN combines the current input trajectory data with the previous memory of the trajectory, which can memorize some important information generated in the process of destination prediction.

(3)It is very easy to embed the deep features extracted by CNN into RNN. In this way, it is easy to show some of the important features hidden in the trajectory data, thus improving the prediction accuracy.

### The process of taxi predicting destination based on RNN

**Fig 7:**
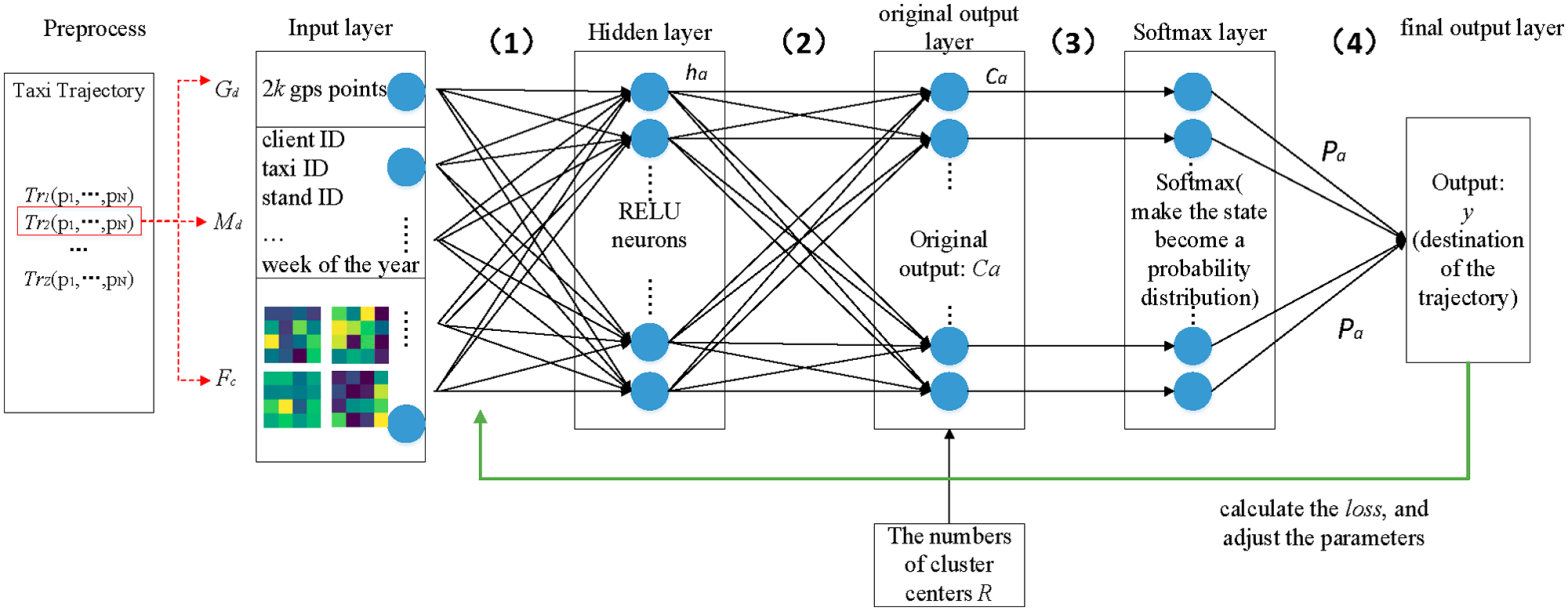
The processes of RNN prediction.

The processes of the taxi destination prediction based on RNN are shown in Fig 7.

In the input layer, each input data include GPS trajectory data *G*_*d*_, trajectory metadata *M*_*d*_ and different combinations of deep features *F*_*c*_. We use *X* to represent the input data of RNN, and the *X* can be written as:

$$\begin{array}{c} X=\{x_{1},x_{2},\ldots ,x_{i},\ldots ,x_{Z}\} \end{array}$$ (16)

$$\begin{array}{c} x_{i}=\left(G_{d},M_{d},F_{c}\right) \end{array}$$ (17)

where *Z* is the number of training data, and 1 ≤ *i* ≤ *Z*. Each training data *x*_*i*_ is a direct combination of *G*_*d*_, *M*_*d*_ and *F*_*c*_. The *G*_*d*_ is a fixed-length trajectory sequence consisting of the first *k* GPS points and the last *k* GPS points of the original trajectory, which gives us a total of 2*k* gps points. The *M*_*d*_ contains client ID, taxi ID, stand ID, quarter hour of the day, day of the week and week of the year. The *F*_*c*_ is one-dimensional feature sequence composed of deep features which extracted by CNN.

Process (1) represents the input layer to the hidden layer, and this process can be written as:

$$\begin{array}{c} h_{a}=f\left(w_{h}\cdot x_{i}^{a}+b_{h}\right) \end{array}$$ (18)

where *h*_*a*_ is the state of hidden layer in step *a*. *w*_*h*_ is the connection weight between the input and hidden layers, $x_{i}^{a}$ is the input data in step *a*, *b*_*h*_ is the bias. The activation function *f* is RELU, we add non-linear elements to the model by using RELU.

Process (2) represents the hidden layer to the original output layer, and this process can be written as:

$$\begin{array}{c} c_{a}=f\left(w_{o}\cdot h_{a}+b_{o}\right) \end{array}$$ (19)

where *c*_*a*_ is the original output in step *a*, *w*_*o*_ is the connection weight between the hidden and original output layers, *b*_*o*_ is the bias. The *f* is also RELU.

Process (3) represents using Softmax [28] function to generate a probability distribution of trajectory destination cluster centers *O*_*m*_(1 ≤ *m* ≤ *R*) and this process can be written as:

$$\begin{array}{c} p_{a}=\frac{c_{a}}{\sum_{j=1}^{R}c_{j}} \end{array}$$ (20)

where *R* is the number of trajectory destination cluster centers by using Meanshift [29].

Process (4) represents the sum of multiplies the corresponding elements between trajectory destination cluster centers and probability distribution, and this process can be written as:

$$\begin{array}{c} y=\sum_{m=1}^{R}O_{m}⊗p_{a} \end{array}$$ (21)

where ⊗ represents the multiplies of the corresponding elements, *O*_*m*_ is the each cluster center point, *p*_*a*_ is the probability distribution in step *a*, *y* is the final output and represents the predict point corresponding to the input trajectory.

After an iteration, according to the cross entropy between *y* and the real destination *y*_*o*_ to determine the loss function, using the loss function to update parameters, and this process can be written as:

$$\begin{array}{c} loss=-[y_{o}\ln y+\left(1-y_{o}\right)\ln\left(1-y\right)] \end{array}$$ (22)

After updating the parameters, the training process will loop until the number of iterations is completed or the loss is small enough. After the model training is completed, the test trajectory data with its features are input into the RNN to predict the destination.

In order to prevent overfitting, we adopt dropout [30] in the processes of training. Dropout make each hidden unit randomly omitted from the network with a probability of 0.5. By this method, the training speed can be accelerated, the robustness of the MTDP-FD can be improved, and the prediction results can be more stable. MTDP-FD takes the deep features as the supplement or enhancement of the trajectory features, and combines with the trajectory data as the input of RNN to improve the prediction accuracy.

### MTDP-FD algorithm

The MTDP-FD algorithm is shown as Algorithm 1:

**Algorithm 1** MTDP-FD algorithm

**Input**: trajectory train data *S*_1_, trajectory test data *S*_2_, trajectory metadata *M*_*d*_;

**Output**: the predicted destination *y*_*p*_ corresponding to each trajectory in *S*_2_;

1: **for** each trajectory (*Tr*)_*i*_ in *S*_1_ **do**

2:  *G*_*d*_ ← take first *k* and last *k* points from (*Tr*)_*i*_;

3:  *I*_*s*_ ← transform (*Tr*)_*i*_ to trajectory image;

4:  *I*_*fh*_, *I*_*fl*_, *I*_*fi*_ ← generate frequency domain representation with frequency domain processing for *I*_*s*_;

5:  *F*_*c*_ ← extract features from *I*_*s*_, *I*_*fh*_, *I*_*fl*_, *I*_*fi*_ and combine features;

6:  *x*_*i*_ = (*G*_*d*_, *M*_*d*_, *F*_*c*_);

7:  Append *x*_*i*_ to *X* to generate the input of RNN;

8: **end for**

9: *O*_*m*_ ← use Meanshift to cluster each trajectory destination in *S*_1_;

10: Initialize the RNN parameters *w*_*h*_, *b*_*h*_, *w*_*o*_, *b*_*o*_;

11: Set number of iterations *n*_*s*_ = 0, iterate terminations *n*_*e*_, everytime training samples *b*_*s*_;

12: $x_{i}^{a}$ ← randomly select *b*_*s*_ *x*_*i*_ from *X*;

13: $h_{a}=f\left(w_{h}\cdot x_{i}^{a}+b_{h}\right)$;

14: *c*_*a*_ = *f*(*w*_*o*_ · *h*_*a*_ + *b*_*o*_);

15: $p_{a}=\frac{c_{a}}{\sum_{j=1}^{R}c_{j}}$;

16: $y=\sum_{m=1}^{R}O_{m}⊗p_{a}$;

17: *loss* = −[*y*_*o*_ ln *y* + (1 − *y*_*o*_) ln (1 − *y*)];// use *loss* to update parameters;

18: *n*_*s*_ = *n*_*s*_ + *b*_*s*_;// each sample is iterated once;

19: **if** *n*_*s*_ ≥ *n*_*e*_ or *loss* is small enough **then**

20:  end trainging;

21: **else**

22:  skip step 12 and continue training;

23: **end if**

24: **for** each trajectory (*Tr*)_*i*_ in *S*_2_ **do**

25:  *y*_*p*_ ← MTDP-FD((*Tr*)_*i*_, *M*_*d*_);

26: **end for**

## Experiments and analysis

### Dataset and environment

The experiment takes the Porto taxi trajectory data [31]. It contains the trajectory data for 442 taxis collected in Porto (Portugal) from July 1, 2013 to June 30, 2014, about one million trajectories. Some of the trajectories may contain less GPS points because the complexity of the surrounding environment, so in order to guarantee the quality of experimental data, we remove the trajectory which contains less GPS points by preprocessing. Trajectory data include GPS points set and trajectory metadata, and trajectory metadata contains client ID, taxi ID, stand ID, quarter hour of the day, day of the week and week of the year. We randomly extract 80% trajectories in the dataset as the training dataset of CNN and RNN, the remaining 20% trajectories as the testing dataset of CNN and RNN. For CNN, the training and testing trajectories in the dataset are transformed into trajectory images.

And In order to transform the trajectory data to the trajectory image as accurately as possible, we take *T* = 60*s*, *W*_*m*_ = *H*_*m*_ = 40, *W*_*p*_ = *H*_*p*_ = 0.2 in transforming trajectory data into trajectory image [22, 23]. In order to obtain effective deep features, we take *C*_*len*_ = 5, *P*_*len*_ = 2 in extracting deep features, so the size of *S*_*pooling*3_ is 4 × 4. In order to keep the beginning and end information of trajectories and reduce the amount of calculation, we take *k* = 5 in forming fixed-length trajectory sequence, so the *G*_*d*_ contains 2*k*(10) gps points.

The experimental program is written in Python2.7 and uses the deep learning framework Tensorflow1.1.0. The experimental operating system uses Ubuntu16.04. Experimental hardware environment is shown as follows: CPU quad-core, Core i5 processor 2.3GHz, memory is 8GB.

### Baseline and metric

We use Average Distance Error (ADE) and Prediction Accuracy (PA) to evaluate the effect of the MTDP-FD algorithm.

ADE: The average distance error (ADE) is calculated by averaging the distance between the point predicted for each trajectory and the true destination point.

PA: The distance between predicted destination and true destination within 500 meters is considered accurate. PA is the ratio of the accurate quantity to the total quantity in the test data.

Then, we compare MTDP-FD with the RNN [19] and the T-CONV [24] prediction algorithm.

We combine different features and data to find out which combination is the best way to improve the prediction accuracy, and the combination ways are described as follows:

(1)GTO: *G*_*d*_, *M*_*d*_, *F*_*o*_.

(2)GTOH: *G*_*d*_, *M*_*d*_, *F*_*o*_, *F*_*h*_.

(3)GTOL: *G*_*d*_, *M*_*d*_, *F*_*o*_, *F*_*l*_.

(4)GTOI: *G*_*d*_, *M*_*d*_, *F*_*o*_ *F*_*i*_.

(5)GTOHL: *G*_*d*_, *M*_*d*_, *F*_*o*_, *F*_*h*_, *F*_*l*_.

(6)GTOHI: *G*_*d*_, *M*_*d*_, *F*_*o*_, *F*_*h*_, *F*_*i*_.

(7)GTOLI: *G*_*d*_, *M*_*d*_, *F*_*o*_, *F*_*l*_, *F*_*i*_.

(8)GTHLI: *G*_*d*_, *M*_*d*_, *F*_*h*_, *F*_*l*_, *F*_*i*_.

(9)GTOHLI: *G*_*d*_, *M*_*d*_, *F*_*o*_, *F*_*h*_, *F*_*l*_, *F*_*i*_.

Since we take the frequency features as the supplement or enhancement of the spatial features, we observe the influence of frequency domain features on the basis of GTO.

### Sensibility of filtering parameters

**Fig 8:**
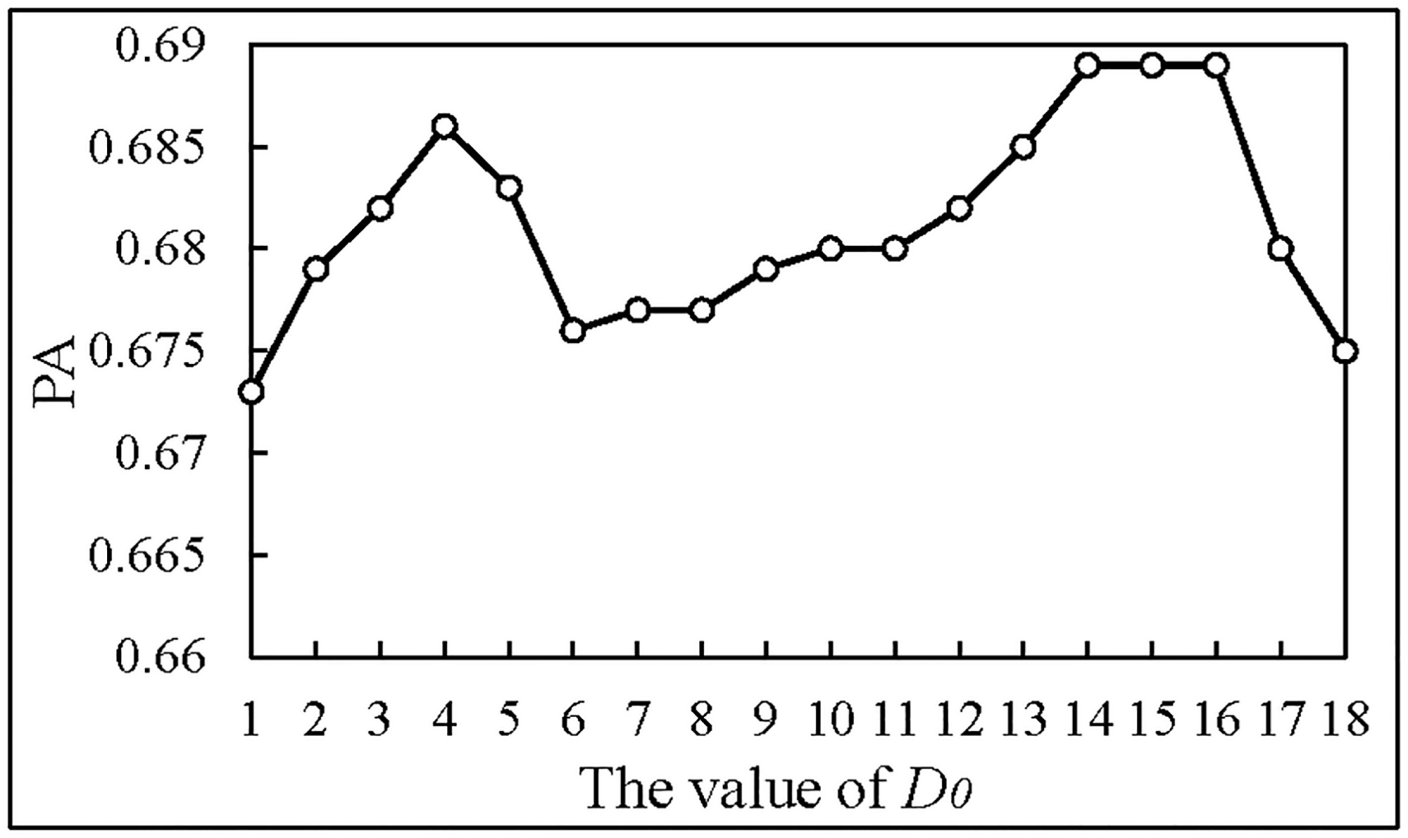
The prediction accuracy of different *D*_0_.

Before MTDP-FD starts large-scale training and prediction, we must determine the parameters of filtering the frequency domain matrix firstly, that is, the values of *D*_0_, *D*_1_, *D*_2_, *D*_3_ in Eqs 7, 8 and 9. And we compare the prediction accuracy by selecting different filtering parameters. In order to determine the parameters quickly and accurately, we record the prediction accuracy of different filtering parameters when the 300 iterations is completed.

(1)We use GTOH as the input of RNN to determine the parameters of high-pass filter, and the result is shown in Fig 8.

**Fig 9:**
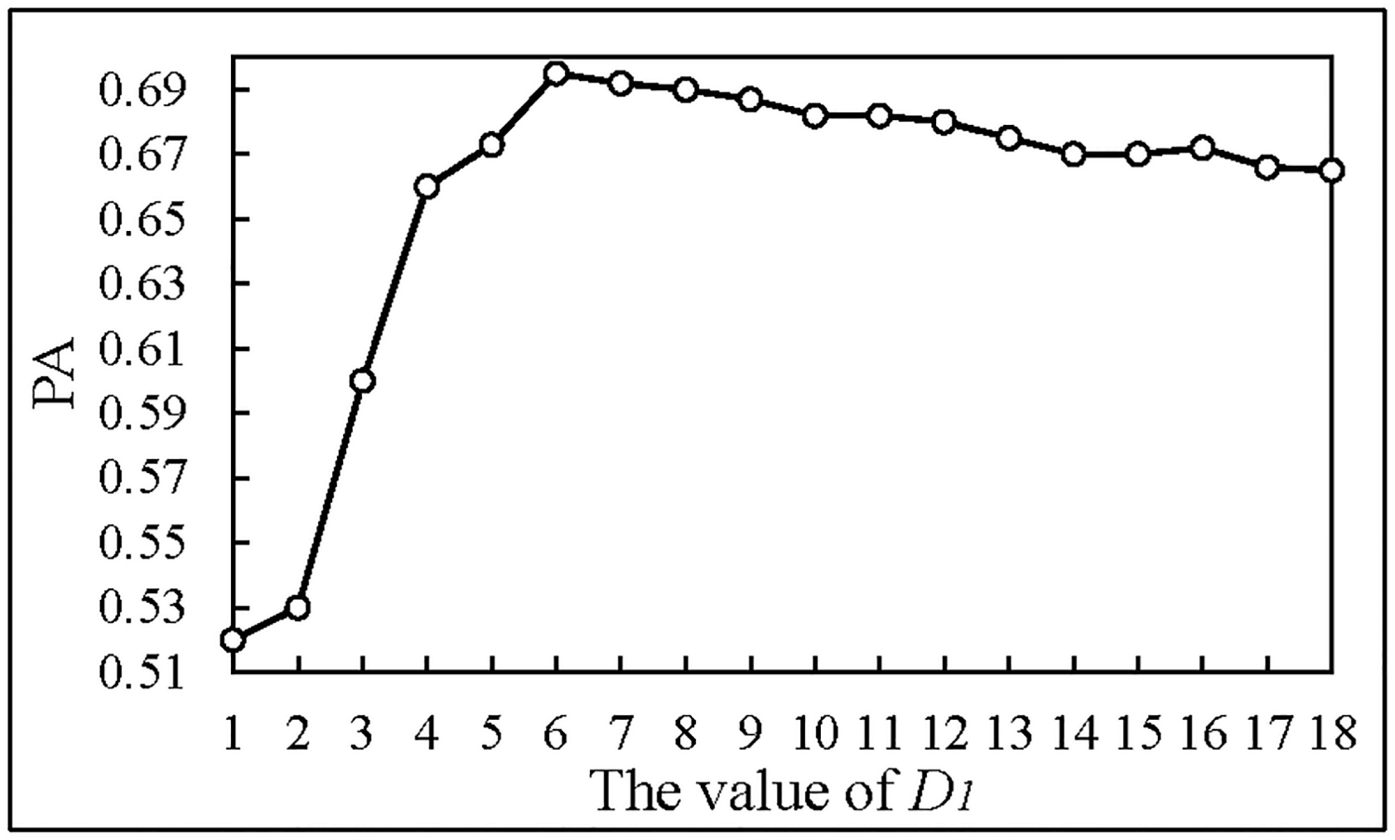
The prediction accuracy of different *D*_1_.

In Fig 8, when the value of *D*_0_ is 14, 15 and 16, the prediction accuracy is higher. So, for compromise, we take 15 as the parameters of the high-pass filter to process images.

(2)We use GTOL as the input of RNN to determine the parameters of low-pass filter, and the result is shown in Fig 9.

**Fig 10:**
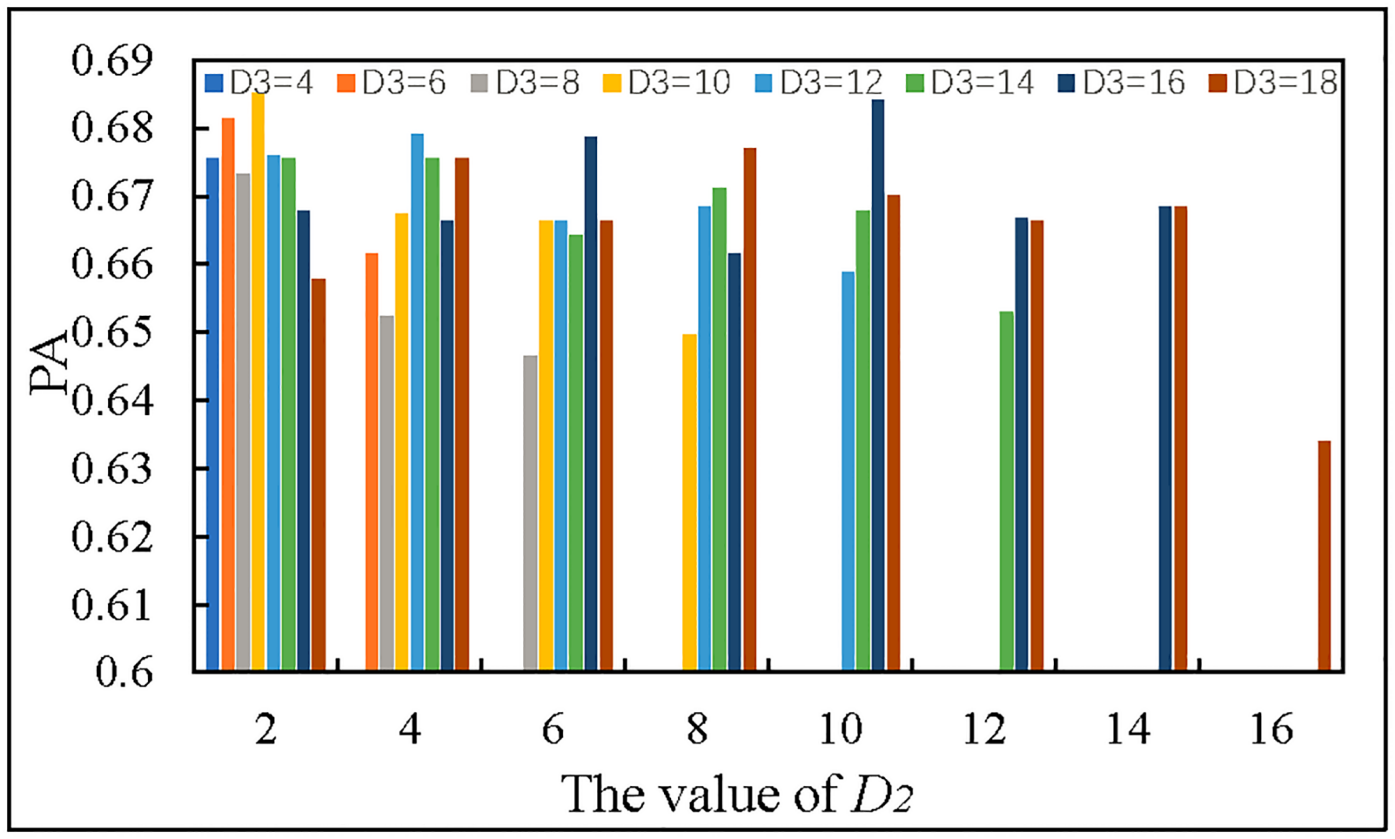
The prediction accuracy of different *D*_2_ and *D*_3_.

In Fig 9, when the value of *D*_1_ is 6, the prediction accuracy is higher. Therefore, we take 6 as the parameter of the low-pass filter to process images.

(3)We use GTOI as the input of RNN to determine the parameters of band-pass filter, and the result is shown in Fig 10.

In Fig 10, *D*_2_ must be less than *D*_3_, when the value of *D*_2_ is 2 and the value of *D*_3_ is 10, the prediction accuracy is higher. Therefore, we take *D*_2_ = 2 and *D*_3_ = 10 as the parameters of the low-pass filter to process images. After determining the filter parameters, MTDP-FD starts large-scale training and prediction.

### Effects of frequency domain features

**Fig 11:**
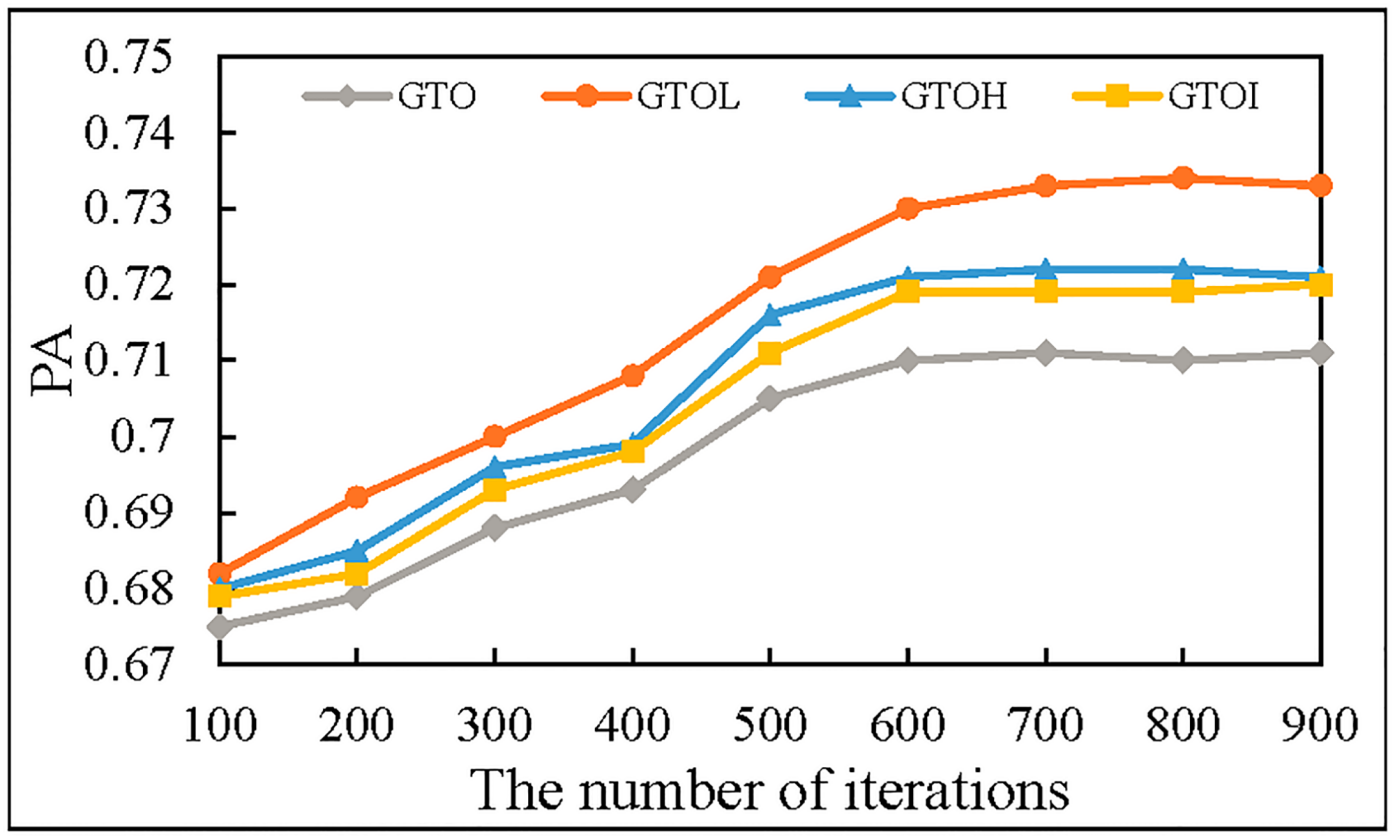
The prediction accuracy of different input.

In order to verify whether the frequency domain features can improve the prediction accuracy, we separately use GTO, GTOH, GTOL and GTOI as the input of MTDP-FD. The results are shown in Fig 11.

In Fig 11, the prediction accuracy of GTOH, GTOL and GTOI is higher than GTO. The above results show that the frequency domain features can be used as a complementary feature in trajectory prediction, and by this way can improve the prediction accuracy. The highest prediction accuracy of GTOL is 0.734, because the low-frequency features highlight the internal relations of the trajectory, thereby alleviating data sparsity and improving the prediction accuracy. The prediction accuracy of GTOH and GTOI is very close.

### Exploring the best features combination

**Fig 12:**
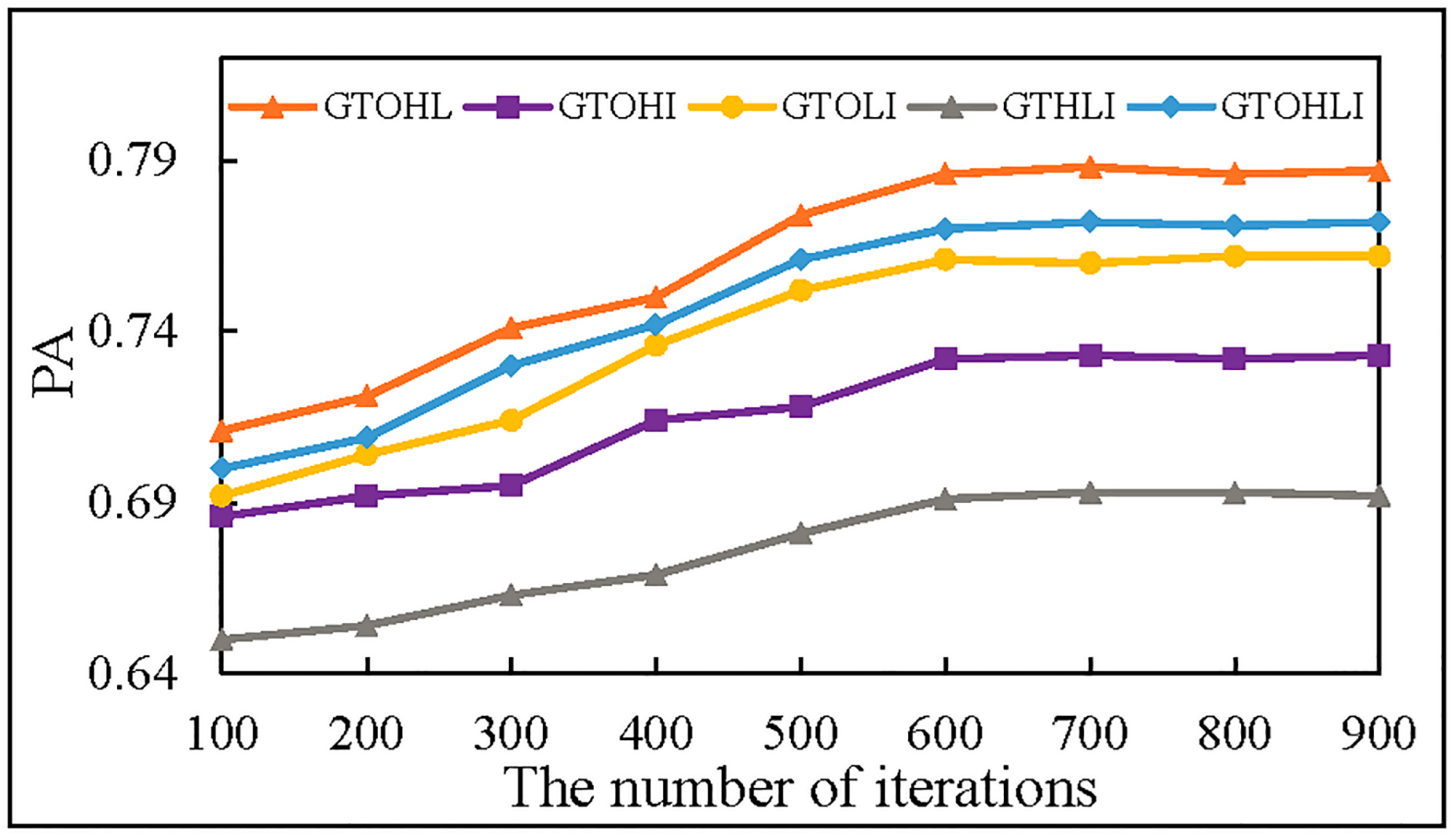
The prediction accuracy of different combinations.

In order to explore the influence of different combinations of features on the prediction accuracy, we separately use GTOHL, GTOHI, GTOLI, GTHLI and GTOHLI as the input of MTDP-FD. The results are shown in Fig 12.

In Fig 12, the prediction accuracy of GTOHL is higher than others. The highest prediction accuracy of GTOHL is 0.788, because GTOHL not only contains the original trajectory image features, but also the high-frequency features show more features of the trajectory edge, and the low-frequency features can show the internal relations of the trajectory. GTOHL is the best combination way to improve the prediction accuracy. GTHLI is the worst combination way, because this way does not contain the original trajectory image features, and it shows that the prediction results are poor only by frequency domain features, frequency domain features should be used as an enhancement or supplement to the original trajectory image features.

### Performance comparison with existing methods

In order to further verify the performance of our method, we compare GTOHL which is the best combination of MTDP-FD with existing methods in average distance error(ADE).

In Table 1, the ADE of MTDP-FD with GTOHL is significantly lower than the RNN and T-CONV prediction methods, and the PA of MTDP-FD is higher than the RNN and T-CONV prediction methods. The RNN prediction method ignores the spatiotemporal information between the trajectory data, thus causing a higher ADE and a lower PA, and the T-CONV prediction method converts data into images to maintain spatiotemporal information between trajectory data, but more noise in the images also make ADE higher and PA lower. MTDP-FD processes the images with frequency domain to reduce the noise which makes the ADE lower and PA higher. Frequency domain features are important enhancement or supplement in the prediction process, improving the prediction accuracy, reducing the average distance error.

**Table 1:** Comparison results.

Prediction methods	ADE(km)	PA(%)
RNN	3.14 [19]	71.06
T-CONV	2.53 [24]	75.62
MTDP-FD(GTOHL)	2.39	78.80

### Features visualization

In order to analyze the deep features easily, we make the images and features visible. There are two advantages to doing this: (1)Observing the relationships between the spatial domain trajectory images, the frequency domain representation of images and the deep features. (2)Observing the differences between the deep features.

**Fig 13:**
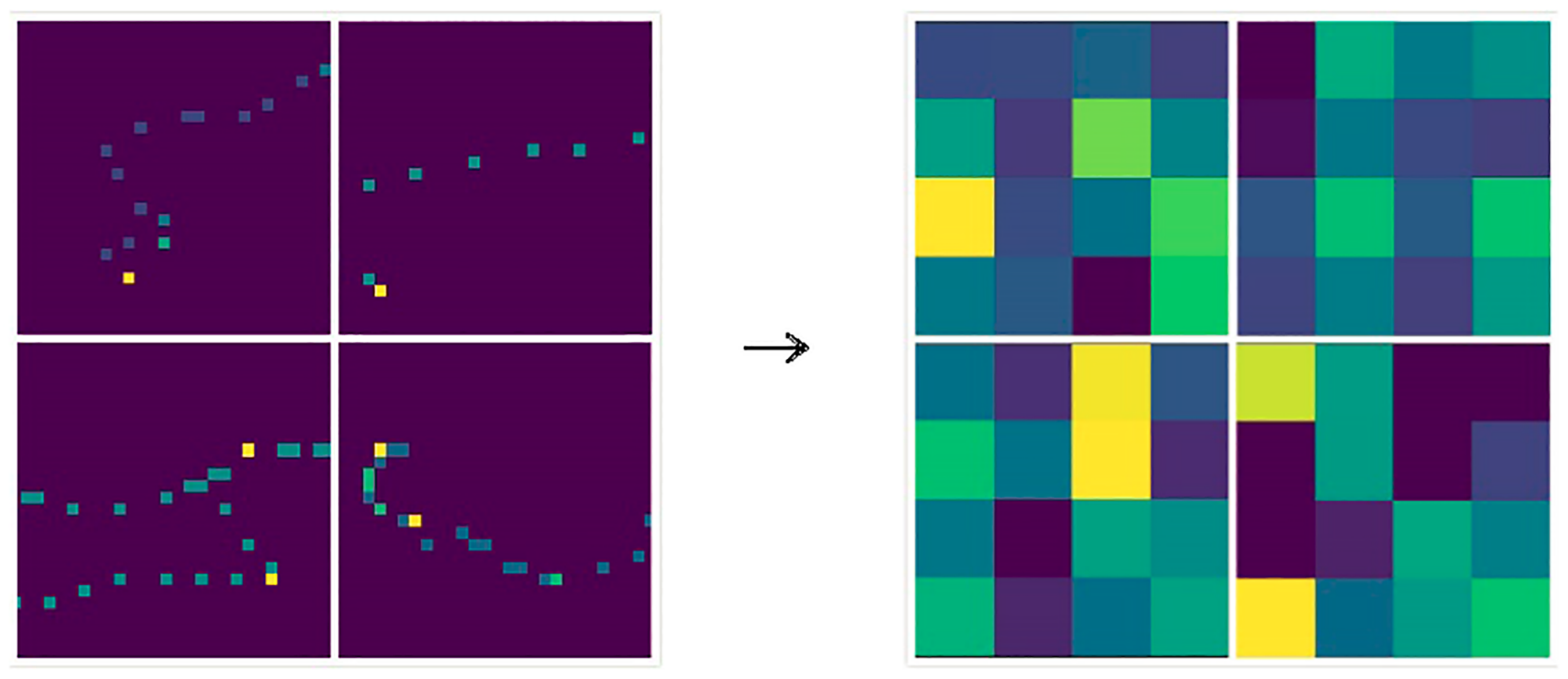
The spatial domain image and its deep features.

**Fig 14:**
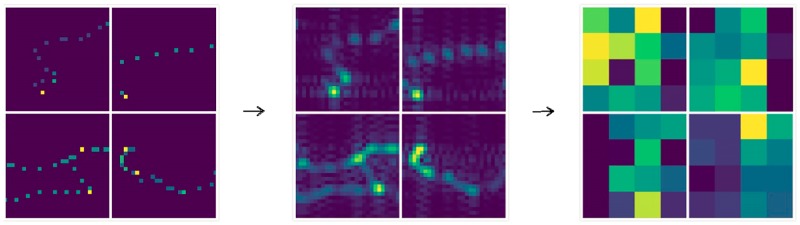
The low-frequency representation of image and its deep features.

**Fig 15:**
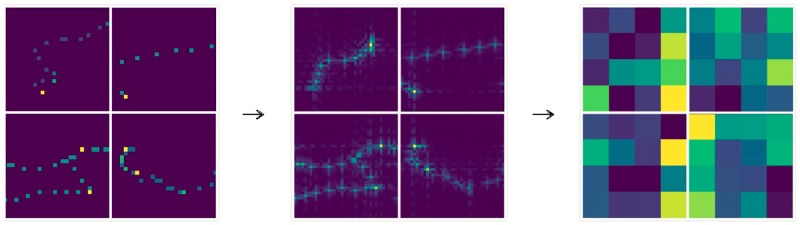
The high-frequency representation of image and its deep features.

**Fig 16:**
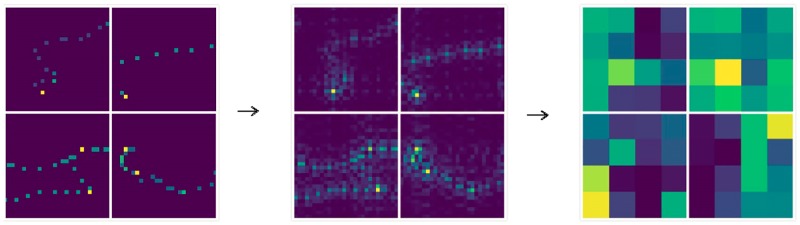
The intermediate-frequency representation of image and its deep features.

In Fig 13, the deep features extracted from the spatial domain trajectory image has become very abstract, and it is difficult to understand all meanings of it. In Fig 14, after low-pass filtering, the edge area of the trajectory becomes very smooth which alleviate the sparsity, and highlights the internal relations of the trajectory. In Fig 15, after high-pass filtering, the edge area of the trajectory is enhanced. The deep features in Figs 14, 15 and 16 are also very abstract, and it is difficult to understand all meanings of them just rely on observation. However, we believe that abstract deep features may contain some important features which spatial domain trajectory images do not have, and these important deep features reflect internal relations of the trajectory, improving the prediction accuracy possibly.

## Conclusion

This paper presents a new approach for taxi destination prediction which called MTDP-FD. The MTDP-FD first applies frequency domain processing to taxi trajectory destination prediction, and the noise of the image is reduced by the frequency domain processing, making the trajectory image more accurate. Experiment shows that the MTDP-FD method could improve the prediction accuracy and the prediction accuracy of GTOHL is higher than other combination ways. However, we just consider the application of high-pass, low-pass and band-pass filter to process image with frequency domain. In addition, the hidden layers of RNN are very sensitive to small perturbations with the increasing amount of data. The perturbations make the error components of RNN enlarged to reduce the prediction accuracy. In the future, we will explore other advanced filters to deal with the image and study how to reduce the effect of perturbations in destination prediction.

## References

[1] Zheng Y. Trajectory Data Mining: An Overview. ACM; 2015.

[2] Hung CC, Peng WC, Lee WC. Clustering and aggregating clues of trajectories for mining trajectory patterns and routes. Vldb Journal. 2015;24(2):169–192. doi: 10.1007/s00778-011-0262-6

[3] Zhang J, Wang FY, Wang K, Lin WH, Xu X, Chen C. Data-Driven Intelligent Transportation Systems: A Survey. IEEE Transactions on Intelligent Transportation Systems. 2011;12(4):1624–1639. doi: 10.1109/TITS.2011.2158001

[4] Tang J, Jiang H, Li Z, Li M, Liu F, Wang Y. A Two-Layer Model for Taxi Customer Searching Behaviors Using GPS Trajectory Data. IEEE Transactions on Intelligent Transportation Systems. 2016;17(11):3318–3324. doi: 10.1109/TITS.2016.2544140

[5] Tang J, Zhang S, Chen X, Liu F, Zou Y. Taxi trips distribution modeling based on Entropy-Maximizing theory: A case study in Harbin city-China. Physica A Statistical Mechanics & Its Applications. 2018;493:430–443. doi: 10.1016/j.physa.2017.11.114

[6] Tang J, Zhang S, Zou Y, Liu F. An adaptive map-matching algorithm based on hierarchical fuzzy system from vehicular GPS data. Plos One. 2017;12(12):e0188796 doi: 10.1371/journal.pone.0188796 29206866 10.1371/journal.pone.0188796 PMC5716534

[7] Tang J, Liu F, Wang Y, Wang H. Uncovering urban human mobility from large scale taxi GPS data. Physica A Statistical Mechanics & Its Applications. 2015;438:140–153. doi: 10.1016/j.physa.2015.06.032

[8] Zhang S, Tang J, Wang H, Wang Y, An S. Revealing intra-urban travel patterns and service ranges from taxi trajectories. Journal of Transport Geography. 2017;61:72–86. doi: 10.1016/j.jtrangeo.2017.04.009

[9] Karlaftis MG, Vlahogianni EI. Statistical methods versus neural networks in transportation research: Differences, similarities and some insights. Transportation Research Part C Emerging Technologies. 2011;19(3):387–399. doi: 10.1016/j.trc.2010.10.004

[10] Xue AY, Zhang R, Zheng Y, Xie X, Huang J, Xu Z. Destination prediction by sub-trajectory synthesis and privacy protection against such prediction. In: IEEE International Conference on Data Engineering; 2013. p. 254–265.

[11] Xue AY, Qi J, Xie X, Zhang R, Huang J, Li Y. Solving the data sparsity problem in destination prediction. Vldb Journal. 2015;24(2):219–243. doi: 10.1007/s00778-014-0369-7

[12] Zhang L, Liu L, Li W. Sparse Trajectory Prediction Method Based on Entropy Estimation. Ieice Transactions on Information & Systems. 2016;E99.D(6):1474–1481. doi: 10.1587/transinf.2015CBP0001

[13] Zhang L, Fan Q, Li W, Liang Z, Zhang G, Luo T. Entropy-Based Sparse Trajectories Prediction Enhanced by Matrix Factorization. Ieice Transactions on Information & Systems. 2017;100(9):2215–2218. doi: 10.1587/transinf.2017EDL8066

[14] Besse PC, Guillouet B, Loubes JM, Royer F. Destination Prediction by Trajectory Distribution Based Model IEEE Transactions on Intelligent Transportation Systems. 2016;.

[15] Zou Y, Zhu X, Zhang Y, Zeng X. A space—time diurnal method for short-term freeway travel time prediction. Transportation Research Part C Emerging Technologies. 2014;43:33–49. doi: 10.1016/j.trc.2013.10.007

[16] Ma X, Tao Z, Wang Y, Yu H, Wang Y. Long short-term memory neural network for traffic speed prediction using remote microwave sensor data. Transportation Research Part C Emerging Technologies. 2015;54:187–197. doi: 10.1016/j.trc.2015.03.014

[17] Ma X, Dai Z, He Z, Ma J, Wang Y, Wang Y. Learning Traffic as Images: A Deep Convolutional Neural Network for Large-Scale Transportation Network Speed Prediction. Sensors. 2017;17(4). 10.3390/s17040818 PMC5422179 28394270

[18] Tang J, Liu F, Zou Y, Zhang W, Wang Y. An Improved Fuzzy Neural Network for Traffic Speed Prediction Considering Periodic Characteristic. IEEE Transactions on Intelligent Transportation Systems. 2017;PP(99):1–11.

[19] De Brébisson A, Simon t, Auvolat A, Vincent P, Bengio Y. Artificial Neural Networks Applied to Taxi Destination Prediction Computer Science. 2015;.

[20] Moore J, Ribeiro B. Using Recurrent Neural Networks in Trajectory Prediction. 2016;.

[21] Endo Y, Nishida K, Toda H, Sawada H. Predicting Destinations from Partial Trajectories Using Recurrent Neural Network. In: Pacific-Asia Conference on Knowledge Discovery and Data Mining; 2017. p. 160–172.

[22] Endo Y, Toda H, Nishida K, Ikedo J. Classifying spatial trajectories using representation learning. International Journal of Data Science & Analytics. 2016;2(3–4):107–117. doi: 10.1007/s41060-016-0014-1

[23] Endo Y, Toda H, Nishida K, Kawanobe A. Deep Feature Extraction from Trajectories for Transportation Mode Estimation. 2016;.

[24] Lv J, Li Q, Wang X. T-CONV: A Convolutional Neural Network For Multi-scale Taxi Trajectory Prediction. 2016;.

[25] Helmy AK, El-Taweel GS. Image segmentation scheme based on SOM—PCNN in frequency domain. Applied Soft Computing. 2016;40:405–415. doi: 10.1016/j.asoc.2015.11.042

[26] Sundararajan D. Image Enhancement in the Frequency Domain. 2017;.

[27] Talathi SS, Vartak A. Improving performance of recurrent neural network with relu nonlinearity Computer Science. 2015;.

[28] Tüske Z, Tahir MA, Schlüter R, Ney H. Integrating Gaussian mixtures into deep neural networks: Softmax layer with hidden variables. In: IEEE International Conference on Acoustics, Speech and Signal Processing; 2015. p. 4285–4289.

[29] He Z, Fan B, Cheng TCE, Wang SY, Tan CH. A mean-shift algorithm for large-scale planar maximal covering location problems. European Journal of Operational Research. 2016;250(1):65–76. doi: 10.1016/j.ejor.2015.09.006

[30] Srivastava N, Hinton G, Krizhevsky A, Sutskever I, Salakhutdinov R. Dropout: a simple way to prevent neural networks from overfitting. Journal of Machine Learning Research. 2014;15(1):1929–1958.

[31] UCI Repository of Machine Learning Databases. Available from: https://archive.ics.uci.edu/ml/datasets/Taxi+Service+Trajectory+-+Prediction+Challenge,+ECML+PKDD+2015.

